# Optimization and validation of multi-state NMR protein structures using structural correlations

**DOI:** 10.1007/s10858-022-00392-2

**Published:** 2022-03-19

**Authors:** Dzmitry Ashkinadze, Harindranath Kadavath, Roland Riek, Peter Güntert

**Affiliations:** 1 Laboratory of Physical Chemistry, ETH Zürich, 8093 Zürich, Switzerland; 2 Institute of Biophysical Chemistry, Center for Biomolecular Magnetic Resonance, Goethe University Frankfurt, Max-von-Laue-Str. 9, 60438 Frankfurt am Main, Germany; 3 Department of Chemistry, Tokyo Metropolitan University, Hachioji, Tokyo Japan

**Keywords:** Multi-state protein structures, Protein structure analysis, Protein structure calculation, eNOE

## Abstract

Recent advances in the field of protein structure determination using liquid-state NMR enable the elucidation of multi-state protein conformations that can provide insight into correlated and non-correlated protein dynamics at atomic resolution. So far, NMR-derived multi-state structures were typically evaluated by means of visual inspection of structure superpositions, target function values that quantify the violation of experimented restraints and root-mean-square deviations that quantify similarity between conformers. As an alternative or complementary approach, we present here the use of a recently introduced structural correlation measure, PDBcor, that quantifies the clustering of protein states as an additional measure for multi-state protein structure analysis. It can be used for various assays including the validation of experimental distance restraints, optimization of the number of protein states, estimation of protein state populations, identification of key distance restraints, NOE network analysis and semiquantitative analysis of the protein correlation network. We present applications for the final quality analysis stages of typical multi-state protein structure calculations.

**Supplementary Information:**

The online version contains supplementary material available at 10.1007/s10858-022-00392-2.

## Introduction

Biological functions of proteins covering target recognition, signal transduction and protein–protein interactions rely on protein dynamics and motion (Ishima and Torchia 2000). Particularly interesting are correlated motions because ligand-induced correlated motion across distant protein sites termed allostery, of which there are several different kinds (Cooper and Dryden 1984; Monnot et al. 1996) appear to be key in many biological systems (Monod et al. 1963).

NMR is a leading technique for experimental studies of dynamics and multi-state structural information of biomolecules because it provides information at atomic resolution and can be measured in aqueous solution (Wüthrich 1990). The probes such as relaxation dispersion, paramagnetic relaxation enhancement (PRE), residual dipolar coupling (RDC), cross-correlated relaxation (CCR), and Nuclear Overhauser Effect (NOE) are of ensemble nature (Clore et al. 1999; Iwahara et al. 2004; Kumar 1985; Riek et al. 1999). For example, the “exact” Nuclear Overhauser Effect (eNOE) yields time-averaged ^1^H-^1^H distances with an accuracy of up to 0.1 Å that can be used to extract multiple coexisting conformations (Orts et al. 2012; Strotz et al. 2017; Vögeli et al. 2016). The eNOEs can be complemented with residual dipolar couplings (RDC) and cross-correlated relaxation (CCR). In summary, robust approaches are now established to determine unbiased multi-state protein structures by NMR.

Recently, we developed the PDBcor software for the analysis of structural correlations in multi-state protein structures (Ashkinadze et al. 2021). Structural correlations highlight correlated motion and represent conserved state-dependent rearrangements shared between distinct protein sites. Prominent structural correlations also allow to identify the state-identity of the modeled protein conformers. Within the PDBcor software, structural correlations indicating correlated motion are evaluated based on distance statistics in the protein bundle. Structural correlations extracted with PDBcor are objective in the sense that they are not based on subjective structure superposition such as principal component analysis (PCA)-based correlations described in previous eNOE works (Vögeli et al. 2012b, 2016). In this manuscript, we demonstrate that structural correlations provide a quantitative parameter alternative to the target function for validating multi-state protein structures.

Conventional NMR structures are calculated following standard procedures using for example the software CYANA (Güntert and Buchner 2015). Protein conformers are fitted to experimental restraints and best solutions corresponding to minimal values of the target function are selected. The main part of the target function responsible for the distance restrains is a weighted sum of the squared violations of the experimental distance restraints (Güntert et al. 1991):

1 $${V}_{d}=\sum_{c=u,l,v}{\omega }_{c}\sum_{(\alpha ,\beta )\epsilon {I}_{c}}{\left({d}_{\alpha \beta }-{b}_{\alpha \beta }\right)}^{2}$$

where ${\omega }_{c}$ are weighting factors for different types *c* = u, l, v of distance restraints, experimental upper limits (*c* = u), experimental lower limits (*c* = l), and steric lower limits (*c* = v), *I*_*c*_ is subset of restraints of type *c* that are currently violated, and ${d}_{\alpha \beta }$ are distances between atoms $\alpha$ and $\beta$ from the calculated protein model that are restrained by experimental limits ${b}_{\alpha \beta }$. In single-state NMR structure calculations, the target function along with the root-mean-square deviation (RMSD) of the bundle of conformers and a list of violated distances in the calculated structure are good indicators to evaluate the quality of the protein structure and thus valuable tools for finding incorrect assignments/distance restraints (Buchner and Güntert 2015; Güntert 2004).

A multi-state NMR structure calculation is based on the assumption that the protein of interest undergoes conformational exchange between different states. Under this assumption, the protein states are fitted to the data including NOEs and RDCs such that an average over these states match best their experimentally measured values. In the case of NOEs, this means that instead of a single distance a set of state-dependent distances is modeled and ensemble-averaged to fit the experimental distance restraints:

2 $${d}_{\alpha \beta }^{*}={\left(\frac{1}{N}\sum_{i=1}^{N}{\left({d}_{\alpha \beta }^{i}\right)}^{-6}\right)}^{-1/6}$$

where $N$ is the number of equally populated protein states and ${d}_{\alpha \beta }^{i}$ is the distance between atoms $\alpha$ and $\beta$ in state *i*. In addition to these ensemble-averaged distance restraints, multi-state structure calculations with CYANA include weak bundling restraints in order to keep the individual structural states together in space, as far as permitted by the experimental restraints (Vögeli et al. 2012a).

## Methods

### PDBcor algorithm

The recently introduced PDBcor algorithm (Ashkinadze et al. 2021) is based on statistics of interresidual distances in the structural ensemble. Since protein ensembles in the case of the WW domain were generated with the assumption of two conformational states, we expect that interresidual protein distances extracted from the NMR ensemble will group into 2 distinct clusters. According to this assumption, the protein conformers were repeatedly (*L* times, where *L* is number of protein residues) clustered in 2 clusters by a Gaussian Mixture Model algorithm based on the distances from a selected residue to all other protein residues. This yields a set of residue-specific conformer clusterings. These clusterings are then compared to each by calculating their mutual information, measured in bits,

3 $$I\left( {X,Y} \right) = \sum\limits_{{x,y = 1}}^{N} {p\left( {x,y} \right){\text{log}}\frac{{p\left( {x,y} \right)}}{{p\left( x \right)p\left( y \right)}}}$$

where $x$ and $y$ are cluster labels of clustering vectors $X$ and $Y$ with probabilities $p\left(x\right)=p\left(X=x\right)$, $p\left(y\right)=p\left(Y=y\right)$ and joint probability $p\left(x,y\right)=p\left(X=x,Y=y\right)$. The absolute value of the mutual information indicates how much the conformer clustering of one residue can tell us about the conformer clustering of another residue. The mutual information is further adjusted to obtain a value of approximately zero for random clusterings. The pairwise adjusted mutual information values form a matrix of correlation values for all residue pairs $A$ that can be visualized in form of a heat map (Ashkinadze et al. 2021). Residues with high average structural correlations to other residues acts as key residues and the optimal global clustering of the protein conformers is set to the clustering vector of such a key residue. Alternatively, the structural correlation matrix *A* can be averaged to a single number, the structural correlation parameter *μ*, that will be used extensively in this paper:

4 $$\mu =\frac{1}{{L}^{2}}\sum_{i,j=1}^{L}{A}_{ij}$$

where the matrix element ${A}_{ij}$ is the adjusted mutual information between residues $i$ and $j$. The structural correlation value *μ* shows how much, on average, the state-identity of the conformers calculated from the distances collected from one residue tells us about the state-identity of the conformers calculated from the distances collected from another residue. The structural correlation parameter $\mu$ is in the range between 0 (absence of correlated motion in the protein ensemble) and 1 (perfect correlation between protein conformers), if it is possible to unambiguously identify the protein states from the calculated protein conformers. Intermediate structural correlation values can be used to quantify the clustering separation between protein states.

All structural correlations *μ* were extracted using PDBcor with default settings (Ashkinadze et al. 2021). Each state of each conformer was provided as a separate protein entity as input for PDBcor. The number of states was set according to the CYANA calculation and the significance threshold that accounts for the influence of random thermal motion was set to 0.5 Å.

### Protein structure calculations

PDBcor was applied to three proteins, for which eNOE-based multi-state structure calculations have been performed previously: the WW domain of PIN1 [PDB ID 6SVC (Strotz et al. 2020)], the protein GB3 [PDB ID 2LUM (Vögeli et al. 2016)] and cyclophilin A [PDB ID 2MZU (Chi et al. 2015)]. The experimental dataset for the WW domain (Strotz et al. 2020) consists of 686 eNOE-derived distance restraints (271 bidirectional ones with exact distances and 415 unidirectional ones with 20% distance uncertainty), and 62 scalar couplings. The experimental dataset for the protein GB3 (Vögeli et al. 2016) consists of 884 eNOE-derived distance restraints, 90 RDCs, and 201 scalar couplings. The experimental dataset for the protein cyclophilin A (Chi et al. 2015) consists of the 3640 eNOE-derived distance restraints, 396 RDCs, and 281 scalar couplings.

Structure calculations for this paper were executed following the established protocol (Chi et al. 2015; Güntert et al. 1997; Vögeli et al. 2012a) using eNORA2 for the spin diffusion correction (Orts et al. 2012; Strotz et al. 2017) and CYANA for structure annealing (Güntert and Buchner 2015; Güntert et al. 1997; Herrmann et al. 2002). Upper and lower limit distance restraints produced by eNORA2, RDCs and scalar coupling restraints were used as input for multi-state structure calculations with CYANA. In each calculation 500 conformers were calculated with simulated annealing using 100,000 torsion angle dynamics steps per conformer. Corresponding heavy atoms from different states were kept together with the help of symmetry restraints in the form of a weak harmonic well potential with a bottom width of 1.2 Å (Güntert et al. 1997; Vögeli et al. 2012a). The 20 best conformers with the lowest final target function values were selected for structural correlation analysis.

## Results

### On structural correlations

Due to the ensemble-averaged nature of the NMR probes including the eNOE (Vögeli et al. 2016) discussed above, multi-state NMR structure determination may yield structural correlations $\mu$ to be defined, detected, and evaluated. In this paragraph a short review on the nature of the structural correlation value $\mu$, the relationship between experimental restraints and structural correlations, and the detection of structural correlations with the use of the software PDBcor (Ashkinadze et al. 2021) are given, using the WW domain of Pin1 as an example (Strotz et al. 2020).

**Fig. 1:**
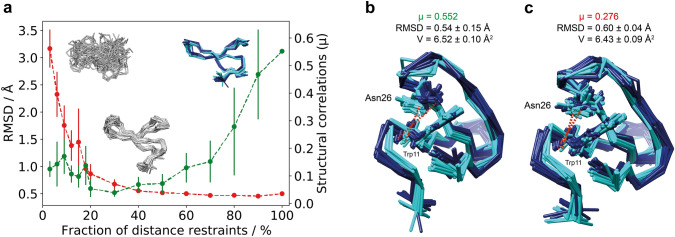
Demonstration of the use of structural correlations on the example of the Pin1 WW domain. Two-state structure calculations were performed for subsets comprising 3–90% of all experimental distance restraints. **a** Mean values and standard deviations of the ensemble RMSD (red) and structural correlations μ (green) as a function of the NOE network density. Two-state structure bundles calculated with 3%, 20%, and 90% of the distance restraints are visualized. **b** WW-domain structure bundle calculated using the full set of distance restraints and colored according to the optimal two-state clustering deduced with PDBcor. **c** Same as **b**, except that one distance restraint of 3.85 Å connecting H of Trp11 and HB2 of Asn26 was excluded from the structure calculation. This distance restraint is depicted by red dotted lines in both bundles

For this purpose, random subsets comprising between 3 and 90% of all 686 assigned experimental eNOE distance restraints were used as input for structure calculations at different levels of the NOE network density. Each experiment was repeated 10 times, always with a new random fraction of the dataset. The resulting structure bundles were then evaluated for the ensemble RMSD and structural correlations as shown in Fig. 1a. A steep descent of the RMSD in the range from 3 to 20% of the eNOEs highlights the approach to the minimally required NOE network density for successfully determining the correct protein fold (structure bundles calculated with 3% and 20% of the complete eNOE dataset are depicted in Fig. 1a). In the context of a two-state structure calculation the finding of the average protein fold means that state-dependent modeled distances ${d}_{\alpha \beta }^{i}$ are not separable and fluctuate around the experimental ensemble-averaged NOE distances reminiscent of a single-state structure calculation. With further increase of the NOE network density the RMSD approaches a value of about 0.5 Å. The protein structure does not converge to a single conformation due to the ensemble-averaged nature of the distance restraints collected from eNOEs. In contrast, it is possible to differentiate between structural states that indicate the presence of motion including correlated protein motion. The PDBcor software (Ashkinadze et al. 2021) can extract such non-random correlated motion from the protein ensemble. The RMSD decrease in Fig. 1a showcases the acquisition of the protein fold, whereas growth of structural correlations detected with PDBcor portrays the protein state separation.

Structural correlations $\mu$ of the calculated WW-domain protein ensembles in the range from 20 to 100% of the input original eNOE dataset steadily grow from 0 up to roughly 0.55 as shown in Fig. 1a. A two-state protein structure calculated from the full NOE dataset is also shown in Fig. 1a. The increasing NOE network density allows not only to find the correct fold, but also to separate two protein states from each other. In the context of the two-state structure calculation, the correct finding of protein states means that modeled state-dependent distances ${d}_{\alpha \beta }^{i}$ become statistically separable. Moreover, it implies that it is possible to separate protein states at distances greater than that of a single NOE with the help of the network effect. As state-specific information is crucial for multi-state NMR protein structure calculations, structural correlations *μ* that probe protein state separation provide an alternative parameter for monitoring and optimization of structure calculation conditions that is complementary to the target function value.

Structural correlations *μ* obtained by the software PDBcor can also be used for the validation of individual distance restraints in protein structure refinement. For multi-state structure calculations, the target function loses its prominent role in finding erroneous restraints, because their impact may get “dissolved” in the additional degrees of freedom that come along with the multiple states. As an example, a two-state structure of the WW domain was calculated with and without an upper limit distance restraint of 3.85 Å connecting the backbone amide H of Trp11 and HB2 of Asn26 (Fig. 1b, c). Structure bundles clearly indicate that the inclusion of this particular distance restraint affects locally the two-state separation of the side chain of Asn26. Nevertheless, the average target function value of the structure bundle including this distance restraint (*V* = 6.52 Å^2^) does not favor it over the structure bundle lacking it (*V* = 6.43 Å^2^). In contrast to the target function values, the average structural correlation values clearly favor the calculation with this distance restraint (*μ* = 0.552) over the calculation without it (*μ* = 0.276). Therefore, the use of structural correlations from PDBcor is considered advantageous in the refinement stage of multi-state structure calculation.

### Optimization of the number of states

**Fig. 2:**
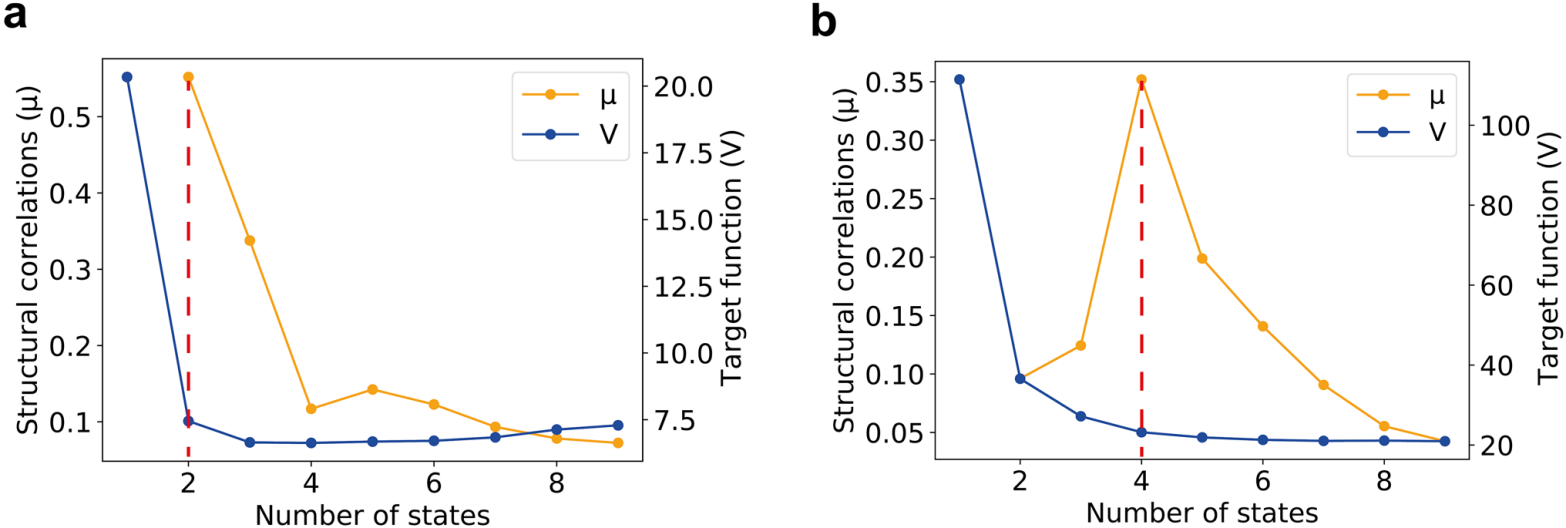
Results of the procedure to determine the optimal number of states for the WW domain (**a**) and the protein GB3 (**b**). The blue line represents the normalized target function and the orange line the average structural correlation as a function of the number of protein states. The correlation value is maximal at two states for the WW domain and four states for GB3, as determined previously on the basis of the normalized target function values. Nevertheless, structural correlation values are easier to interpret due to their prominent maximum at the optimal number of states

For a multi-state structure determination, the number of protein states that can be resolved meaningfully by the experimental restraints must be determined. The established procedure uses the target function decrease with the number of states calculated. The number of protein states is assessed by calculating protein ensembles with 1 to 9 states. The optimal number of states is then set according to the multi-state ensemble that achieves a minimum of the normalized target function or in other words to the minimum required number of states necessary to explain the experimental data. This is illustrated here for two previously reported model proteins, the WW domain of PIN1, yielding a two-state system, and GB3, yielding a four-state system, by multi-state calculations with previously reported procedures using CYANA and the published experimental restraints (Strotz et al. 2020; Vögeli et al. 2016) (Fig. 2). We evaluated the ensembles with 1 to 9 states in terms of structural correlations using PDBcor. Structural correlations were calculated only for two or more states since at least two states are required for the meaningful extraction of structural correlations. As it is clearly visible in Fig. 2, the normalized target function reaches a minimum at the reported number of states in both cases and levels off with increasing number of states (Strotz et al. 2020; Vögeli et al. 2016). As opposed to the target function, structural correlations of calculated structures do not plateau but show a maximum at the correct number of states. Assuming that biologically a protein behaves as an *N*-state system, structural correlations are expected to increase approaching the *N* states with increasing model complexity that is able to properly fit the experimental data and counter underfitting. In addition, if less than the existing number of states are included in the structure calculation, violations of the experimental restraints occur (manifested by the higher target function values in Fig. 2), which results in deteriorations of the structures that are likely irregularly distributed both structures and states and thereby yield a loss of correlations. Structural correlation values decrease thereafter with additional modeled states as extra states that start to fuse with existing states, making them statistically inseparable, and lead to overfitting. Hence, structural correlations provide an alternative method to determine the optimal number of states for multi-state structure calculation that can give more clear-cut results than the conventional target function-based analysis and are best used in concert with each other.

### Estimation of protein state populations

**Fig. 3:**
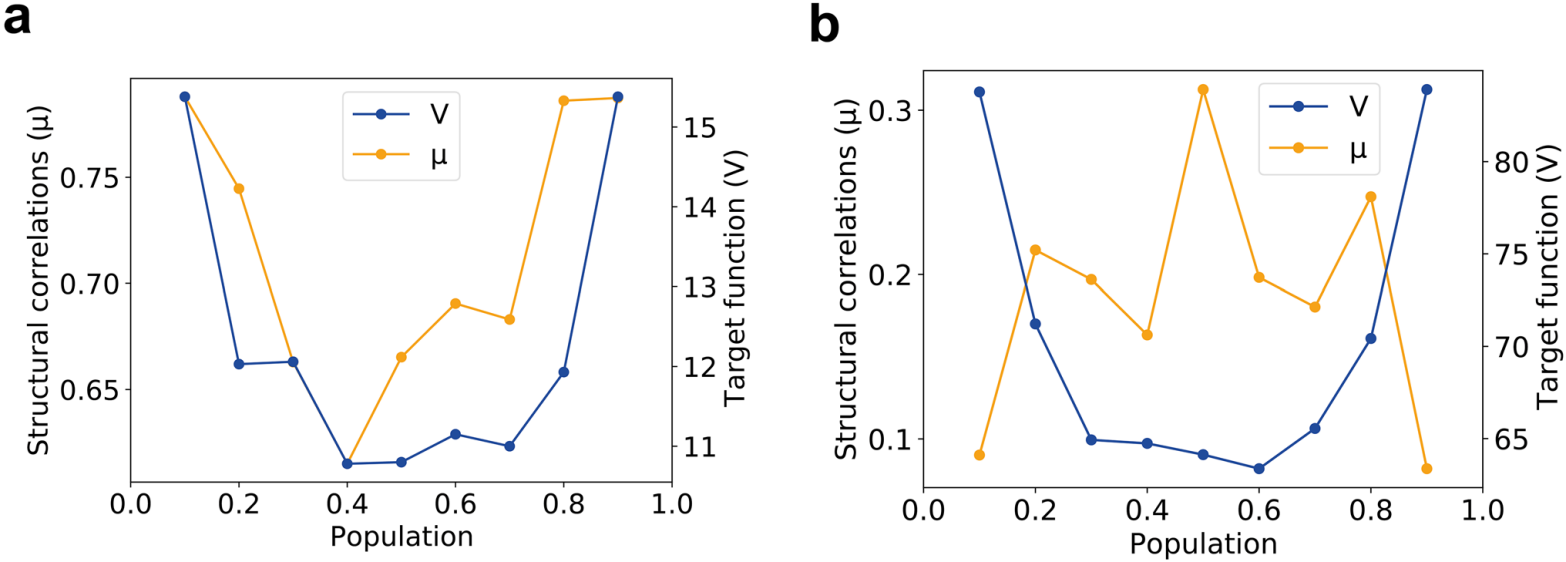
Results of population estimation studies for the WW domain (**a**) and protein cyclophilin A (**b**). The blue line corresponds to the normalized target function and the orange line to the average structural correlation as a function of the protein state A population

The large majority of documented multi-state protein structures feature a two-state model (Bai and Englander 1996). For such two-state models populations of individual states can be evaluated empirically by conducting a series of ten-state CYANA structure calculations in which the 10 individual states are separated in two controlled groups A and B (Strotz et al. 2020). Protein states in each group are tightly bound to each other. By varying the size of group A from 1 up to 9 conformers, we can simulate a protein structure with population of state A rising from 10 up to 90%. In the established procedure, optimal protein state populations are determined according to the minimum of the normalized target function (Vögeli et al. 2016). Here, we present an estimation of protein state populations for two previously reported model proteins, the WW domain of PIN1 and cyclophilin A. In addition, for both systems protein ensembles calculated with varying population parameters were evaluated in terms of normalized target function values and structural correlations using PDBcor (Fig. 3). For the latter, two protein conformations representing both protein states were selected from each ten-state structure calculation and used as input to PDBcor making population analysis equivalent to the analysis of a series of two-state protein structures. Figure 3 shows that the target function approaches its minimum in range of 40–60% for both systems. Structural correlations for cyclophilin A exhibit a maximum at a state population of 50% with a slight shoulder at 20% and (equivalently) 80%. According to these observations the two protein states of cyclophilin A are populated equally or 20/80 judging by the structural correlation shoulder. Despite target function minimum at 40% structural correlations of the WW domain show a maximum at 10/90. However, it was also previously reported, that the estimation of protein state populations using the target function appears to be difficult for the WW domain (Strotz et al. 2020). While the structural correlations appear to be an alternative predictor of populations, it remains a difficult task.

### Identification of key distance restraints for structural correlation for validation purposes

**Fig. 4:**
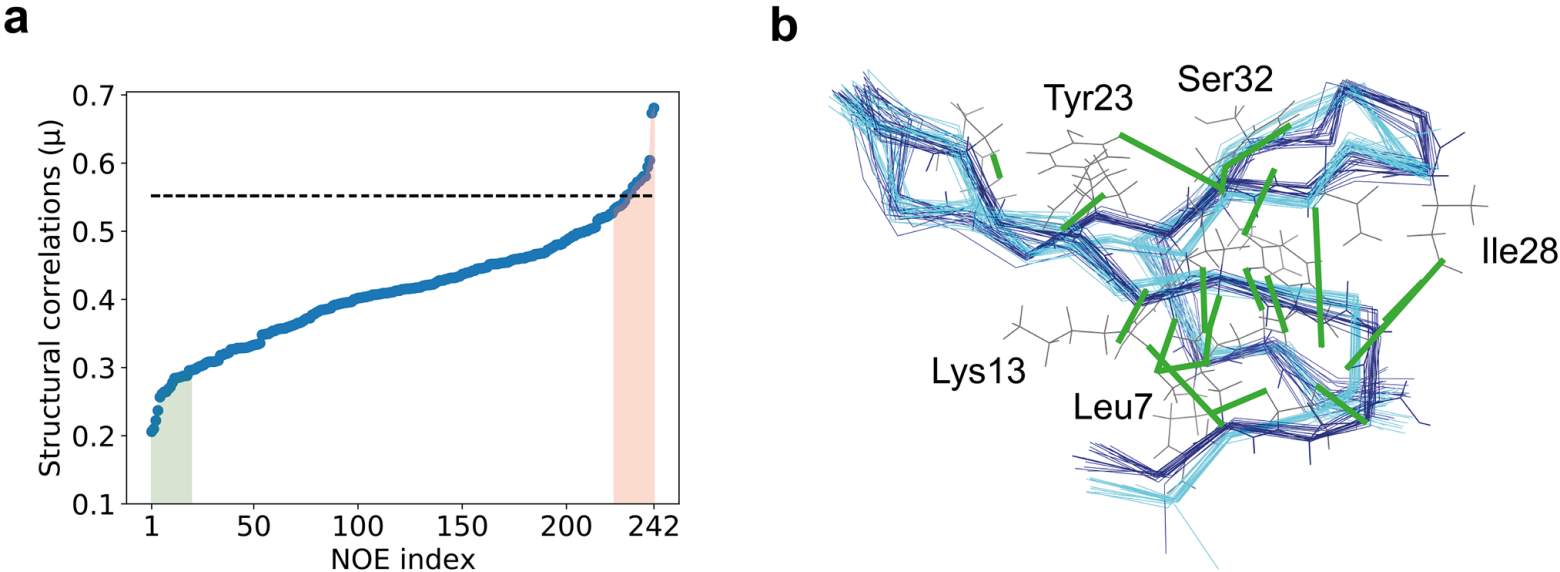
Results of the key distance restraint assay for the WW domain. **a** Long-range distance restraints from the WW domain were sorted according to the average structural correlation value obtained in a two-state structure calculation after their removal. The structural correlation value of the two-state structure obtained with all distance restraints is indicated by the dashed line. Distance restraints corresponding to the twenty highest and twenty lowest structural correlation values are highlighted in red and green, respectively. **b** The twenty eNOEs invoking the biggest allosteric reduction (key eNOEs) are further depicted by green lines on a two-state WW domain structure

In a multi-state structure determination, the identification of key eNOE distance restraints that reveal structural correlations is important in order to check their validity individually by inspection of the NMR spectra and analyses such as the NOE build-up rate quality. In order to find these key restraints individual distance restraints can be evaluated empirically in terms of structural correlations by calculating structures omitting a particular distance restraint. As an example, a complete series of two-state structure calculations missing one particular long-range distance restraint was performed for the previously mentioned WW domain. Subsequently, each calculated bundle was evaluated for structural correlations and distance restraints were sorted in ascending order of the average structural correlation (Fig. 4). A decrease in structural correlation caused by the removal of a particular distance restraint can either indicate that it is a key folding NOE restraint or a NOE restraint orchestrating correlated motion and protein states splitting. On the contrary, a structural correlation increase due to the removal of a particular eNOE could indicate potential structure calculation problems including distance restraint inaccuracy or misassignment. For the example discussed in Fig. 4, the list of eNOEs corresponding to 20 protein ensembles with highest structural correlation values is given in Table S1. Distance restraints whose removal contributed either to the twenty highest or twenty lowest structural correlation values were selected for further evaluation. In Fig. 4b, key NOEs that were mapped onto the 3D structure are concentrated in the WW allosteric site and domain termini (Strotz et al. 2020). Nevertheless, since this approach evaluates contributions of individual NOEs, a possible contribution by distance restraints that are part of a redundant NOE subnetwork might be underestimated. Without these 20 eNOEs neither the two-state structure determination did significantly alter (not shown), nor the correlation got lost (Fig. 4a), which can be attributed to experimental data redundancy.

### Distance range of structural correlations derived from eNOE distance restraints

**Fig. 5:**
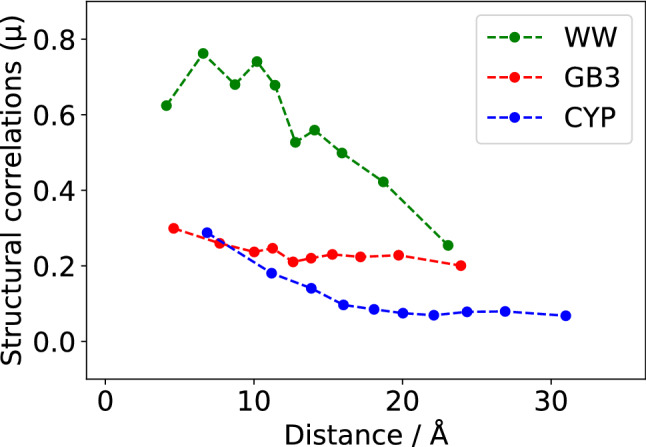
Distance dependence of structural correlations for the WW domain of PIN1 (green), the protein GB3 (red) and cyclophilin A (blue). Structural correlations for the WW domain and cyclophilin A exhibit a steeper decline than for the protein GB3, which makes them more locally correlated than GB3. Significant structural correlation for distances above 5 Å (the maximum range of a single NOE) can only be explained by the effect of the NOE network

We also studied how structural correlations derived from eNOE restraints depend on the distance between residues. Three deposited multi-state protein structure ensembles, including the WW domain of PIN1 (PDB ID 6SVC (Strotz et al. 2020)), the protein GB3 (PDB ID 2LUM (Vögeli et al. 2016)) and cyclophilin A (PDB ID 2MZU (Chi et al. 2015)), were analyzed for structural correlations. For each system, all residue pairs were sorted in ascending order according to their average C^α^–C^α^ distance in the published structures and separated into ten equal groups. Then, the average interresidual distance and the average structural correlation value was calculated for each group and plotted in Fig. 5. Results show that the average structural correlation values decrease with increasing distance between residues as it would be expected for local structural correlations that are limited in their span. Nevertheless, a certain level of structural correlations is retained throughout all distance groups as it would be expected for global structural correlations that are independent of the interresidual distance. Results also clearly indicate that correlated motion spans significantly larger distances than a single NOE (i.e., 5 Å), which can only be attributed to a collective influence of the NOE network.

### Optimization of the CYANA multi-state structure calculation using structural correlations

**Fig. 6:**
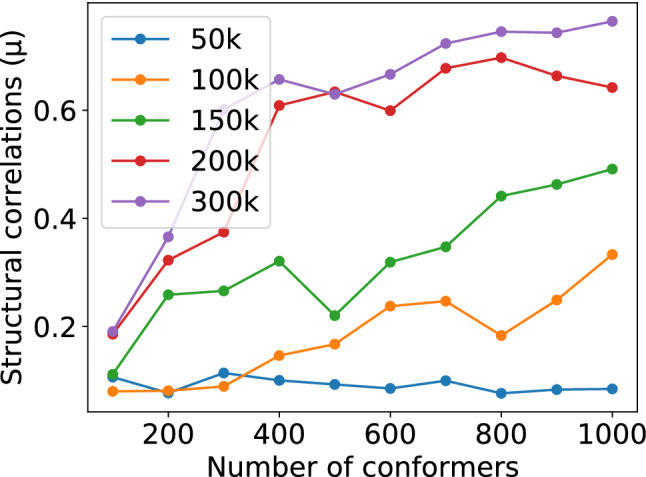
Screening of multi-state structure calculation conditions for the WW domain. Structural correlations of two-state structure bundles indicate that the conventional calculation of 100 conformers with 50,000 torsion angle dynamics steps is not sufficient for convergence. Therefore, the basic structure calculation protocol was adjusted to 500 conformers with 200,000 torsion angle dynamics steps

During the extensive testing of the above validation concepts, we also noticed that an insufficient number of conformers calculated with CYANA and an insufficient number of torsion angle dynamics steps can affect the structural correlation values through a suboptimal sampling by the calculated conformers. In particular, the number of torsion angle dynamics steps can have a major influence as shown in Fig. 6. In order to illustrate the undersampling issue a series of two-state structure calculations of the WW domain were performed varying the number of torsion angle dynamics steps and the number of calculated conformers. Convergence was observed on the basis of structural correlations. The structure calculation convergence results summarized in Fig. 6 indicated an adjustment of the CYANA calculation parameters to 200,000 torsion angle dynamics steps and 500 calculated conformers as optimal conditions for a multi-state structure determination.

## Discussion

NMR-based multi-state structure determination is established (Chi et al. 2015; Güntert et al. 1997; Vögeli et al. 2012a) and has been demonstrated for 4 systems using eNOEs (Chi et al. 2015; Strotz et al. 2020; Vögeli et al. 2016, 2009). The major remaining challenge that we identified in the protocol is the validation of the multi-state structures because the usual approach in standard structure calculations using the target function along with the list of remaining restrained violations (Güntert 2004; Güntert et al. 1991; Güntert and Buchner 2015) appeared not be sufficient to find all erroneous restraints or eNOE build-up curves, requesting detailed extensive manual analysis of individual restraints and NOE build-up fits along with many test calculations resulting in manually adapted, time-consuming and non-standardized procedures.

Here, we demonstrated that the structural correlations obtained with the software PDBcor are an additional tool for the validation of multi-state structure determinations that provides straightforward information on the degree of overdetermination of the system, lists key restraints responsible for the identified structural correlations, and identified the number of states including their approximate populations necessary to fulfill the experimental restraints. Structural correlations are thus an important probe in the refinement stage of a multi-state structure calculation as they are sensitive to the protein state splitting, while the target function and the list of violated experimental restraints are important in earlier steps of the multi-state structure determination (in particular at the single-state and initial two-state structure determination phase). Together they constitute a powerful tool for the validation of NMR-based multi-state structures.

The PDBcor software for the calculation of structural correlations is freely available (https://github.com/dzmitryashkinadze/PDBcor) (Ashkinadze et al. 2021). PDBcor allows the straightforward and objective determination of structural correlations in a given multi-state protein structure. The assays and subroutines performed and demonstrated here together with corresponding examples were deposited at http://www.cyana.org/wiki/index.php/Tutorials and can be straightforwardly adopted to individual systems. Together with the software package CYANA (Güntert and Buchner 2015; Güntert et al. 1997), including the eNORA software (Orts et al. 2012; Strotz et al. 2017) for NOE build-up rate determinations, multi-state structures can be determined efficiently given NOESY cross peak assignments and intensities as an input. With these tools multi-state structures can be determined readily using eNOE restraints. The additional NMR measurement time to acquire several (i.e., 3–4) combined ^15^N,^13^C-resolved [^1^H,^1^H]-NOESY experiments instead of one is only approximately one week in order to obtain a multi-state structure that comprises the correlated dynamics of the protein of interest at atomic resolution and as such a unique quantitative information of presumably high biological relevance that currently no other technique than NMR can produce.

## Supplementary Information

Below is the link to the electronic supplementary material.

Supplementary file1 (PDF 53 KB)

## Data Availability

The PDBcor software for the calculation of structural correlations is freely available at https://github.com/dzmitryashkinadze/PDBcor. Selected assays together with corresponding demonstration examples are freely available at http://www.cyana.org/wiki/index.php/Tutorials.
