# Supplementary material for: Optimization and validation of multi-state NMR protein structures using structural correlations
Source: J Biomol NMR. 2022 Mar 19;76(1-2):39–47. doi: 10.1007/s10858-022-00392-2 (PMC9018667; doi:10.1007/s10858-022-00392-2)
Supplement: Supplementary file 1 — Supplementary file1 (PDF 53 KB) [file 10858_2022_392_MOESM1_ESM.pdf]

Supplementary Information

Optimization and Validation of Multi-state NMR Protein Structures using Structural Correlations

Dzmitry Ashkinadze, Harindranath Kadavath, Roland Riek*, Peter Güntert*

Laboratory of Physical Chemistry, ETH Zürich, Vladimir-Prelog-Weg 2, CH-8093 Zürich, Switzerland
Institute of Biophysical Chemistry, Center for Biomolecular Magnetic Resonance, Goethe University, Frankfurt am Main, 60438 Frankfurt am Main, Germany
Department of Chemistry, Tokyo Metropolitan University, Hachioji, Tokyo 192-0397, Japan

*Correspondence to peter.guentert@phys.chem.ethz.ch and roland.riek@phys.chem.ethz.ch

TABLES

ID	1	2	3	4	5	6	7	8	9	10	11	12	13	14	15	16	17	18	19	20
**Residue 1**	26	7	25	13	16	8	33	23	15	16	12	25	13	15	14	13	11	10	13	24
**Atom 1**	HB2	QD2	HZ	QE	H	HG3	HE2	QE	HG2	HB3	QB	QD	HD2	QB	HG3	HD3	HH2	HA2	QD	HB3
**Residue 2**	31	24	30	24	22	39	37	32	22	23	27	32	24	22	25	24	33	28	22	37
**Atom 2**	QB	QE	HA	QE	HA	QA	HD2	HB3	HA	QD	HD2	HB2	QE	QG2	HA	QE	HB2	HG1	QG2	HD2
**Correlation**	0.532	0.535	0.536	0.537	0.540	0.544	0.553	0.554	0.556	0.565	0.567	0.572	0.573	0.577	0.581	0.581	0.594	0.604	0.673	0.681

Table S1 The 20 eNOEs for the WW domain that yield the highest structural correlation values for a two-state structure bundle calculated without the respective eNOE.
